# Effective Recruitment Strategies Utilized to Examine Dietary Practices of Blacks in New York City in the Midst of the COVID-19 Pandemic

**DOI:** 10.1007/s40615-023-01559-9

**Published:** 2023-03-16

**Authors:** Cicely K. Johnson, May May Leung, Grace X. Ma, Olorunseun O. Ogunwobi

**Affiliations:** 1 Hunter College Center for Cancer Health Disparities Research, Hunter College of the City University of New York, New York, NY USA; 2 Nutrition Program, School of Urban Public Health, Hunter College of the City University of New York, New York, NY USA; 3 Center for Asian Health, Temple University, Philadelphia, PA USA; 4 Department of Biological Sciences, Hunter College of the City University of New York, New York, NY USA

## Abstract

**Background:**

Black Americans have long been considered a hard-to-reach population for research studies, whether quantitative surveys or for clinical research. Studies have explored multiple rationales for why Blacks are hard to reach, and the explanations have included historical mistrust, the need to assess the benefits from participating in research, and the expense of spending time participating in research, among others. What has not been explored is the continuous merging of all individuals who identify as Black, particularly when exploring reasonings for a lower interest in participating in research. This paper addresses this issue by investigating the participation rate of individuals identifying as Black in New York City in a study exploring dietary practices as a predictor of colorectal cancer screening behavior. Participants were asked to self-report screening behavior, intent to screen, and dietary and other lifestyle practices. In this analysis, we discuss the unique experience encountered in recruiting Black American participants to participate in this study, particularly amid a worldwide pandemic of COVID-19.

**Methods:**

The methodology for this study included a systematic review of the literature, a two-part recruitment process, and data analysis. The first part of the recruitment process involved registering individuals who were interested in participating in the study and consented to be contacted and reminded to come to the location where they were recruited on a scheduled date to complete the actual survey. With this part of the recruitment process, we engaged with *n* = 488 Black men and women between November 2019 and February 2020. The second part of the recruitment process utilized availability sampling outside of NYC subway stations and other high traffic areas as well as large community events. We engaged with *n* = 319 individuals. Total engagement with *n* = 807 individuals yielded a sample size for the survey of 504 completed surveys.

**Results:**

Of the total engaged (*n* = 807), 14% declined to participate due to a lack of time, 11% chose not to participate in the study because the incentive was not enough to compensate for their time 0.02% declined due to not trusting institutions conducting research, and 0.03% did not feel comfortable understanding the questions due to a language barrier. We had a sample size of (*n* = 504) of the total 807 individuals engaged.

**Conclusions:**

Recruiting Black Americans into our colorectal cancer study did not prove to be challenging with the two-tiered model of recruitment that involved consistent engagement and having the primary researcher lead this recruitment process. Extracting within race differences is critical in demystifying the conclusion of numerous studies that African Americans specifically are hesitant to participate due to historical mistrust related to tragedies such as the Tuskegee Experiment and numerous other occurrences of African Americans being treated as guinea pigs for the advancement of research. This data contributes knowledge to this field regarding understanding recruitment challenges in the Black population, but further work needs to be conducted. Mistrust in this study primarily came from the individuals engaged in Caribbean neighborhoods, where many expressed more comfort with home remedies and bush doctors when asked about colorectal cancer screening and declined to participate. Innovative communication, qualitative research, and recruitment strategies tailored to the Caribbean population are needed in future studies to address this recruitment challenge that we experienced.

## Background

Lower research participation among Blacks has long been documented, and many studies have studied the reasons for lower participation. Many studies have attributed medical mistrust as being a number one reason for lower participation [1, 2]. Distrust, historical atrocities, time, family/work responsibilities, socioeconomic stressors, and confidentiality have been identified in multiple studies as the major reasons for lower participation among Blacks [2]. Many of these studies however have historically grouped Blacks together and titled the conclusions and articles about African-Americans. Historical atrocities, medical mistrust, and socioeconomic stressors can certainly vary by culture and cultural context. The socioeconomic stressors could also certainly vary by location and a myriad of other antecedent variables such as family structure, family support, employment, and income. While all of that cannot be covered in this article, this is a pivotal staring point to continue exploring how the critical interplay of culture impacts decisions regarding research participation. Presence and interaction of the researcher has also been documented as a salient factor impacting Black participation in research. Qualitative studies have revealed that community members have expressed the desire for researchers to have a presence in the community, noting that investigator engagement that spans beyond the university can have a significant impact on recruitment and retention [3]. Some qualitative studies have explored the significance of race of the researcher on Black participation, and have concluded through participant’s voice that race is secondary to involvement:

“Researchers that are out there in the community, getting to know the people, … spending time with them, aren’t hard into academia. Those are the ones able to do the recruitment, whether they’re White or Black [3].”

Also significant to this study is that previous studies have found that despite commonly discussed barriers to Black participation such as distrust and time playing a significant role, the main obstacle to recruitment was successful recontact [4]. Some researchers engage within the community and do not return to strengthen the relationships that have been made.

The study for which data was being collected, and the recruitment methods were being explored, was examining dietary practices of Blacks in New York City, theorizing that those with healthier dietary practices were potentially more likely to adopt other healthy behaviors like cancer screening. Colon and rectum cancer are among the most commonly diagnosed cancers in the USA. The percentage of older adults who are up to date on core preventive services is low, with solely 25% of adults ages 50–64 and less than half of adults ages 65 years and older, reporting recent attendance to preventive clinical services [5]. An estimated 15 to 40% of individuals who are eligible to be screened for breast, cervical, and colorectal cancer are not up to date with screening [6]. Recent years have shown an increase in emphasis on colorectal cancer prevention and screening. From 2000 to 2015, colorectal cancer screening among adults ages 50 and older increased from 32 to 62% in non-Hispanic Blacks, compared to an increase from 40 to 65% in non-Hispanic whites [7]. A recent study found that Blacks were 30% more likely than whites to be diagnosed with an interval cancer, which occurs after a negative colonoscopy but before the next recommended screening [8], which really calls into question variables such as diet and lifestyle.

In 2018, the American Cancer Society (ACS) released an updated guideline for colorectal cancer screening that changed the recommended starting age for screening to 45 years for people at average risk, versus the previous recommendation of starting at age 50 [9], as new cases of colorectal cancer are occurring at an increasing rate among younger adults. We set out to assess screening behaviors of study participants ages 45 and over, in addition to assessing the intent to screen of those turning age 40 in 2020 (the year of data collection), who would reach the recommended age within 5 years. Colorectal cancer is the third most common cancer in Black men and women, and it is the third-leading cause of cancer death in both Black men and women [10] Among major modifiable factors that increase risk for colorectal cancer included are the high consumption of red or processed meat, moderate to heavy alcohol consumption, and very low intake of fruits and vegetables and whole-grain fiber [10]. As this disease is highly prevalent in Black populations [11] and few studies have assessed the relationship between dietary habits and screening behavior for colorectal cancer specifically among individuals who identify as Black, our recruitment targeted individuals who identify as Black. This paper explores our recruitment process of the Black population in a city as within race diverse as New York City and aims to identify successful recruitment strategies that were utilized. Part of the research team for this study had also previously explored recruitment strategies utilized while working to decrease obesity risk among urban minority preadolescents [12] which provided a great deal of insight for this study.

New York City has a notable mix of cultures, including large Caribbean and African populations. In 2014, the Black non-Hispanic population of New York City numbered 1.89 million, which was more than double the count in any other US city [13]. This unique diversity provides an opportunity for a myriad of novel research studies. Between 2000 and 2010, the number of legal Black African immigrants in the USA nearly doubled, to around one million. These multiethnic groups must be recognized to better understand unique cultural variances. New York is home to the largest Black immigrant population in the country and has been since 2000 [14]. New York City specifically has the largest Black immigrant population—roughly 260,000 Black Jamaican immigrants live in the New York City metro area as of 2019, representing 35% of all foreign-born Jamaicans in the country. As of 2019, the vast majority of Black immigrants live in either the Northeast (1.1 million) or the South [14]. While considering the connections to medical mistrust, for example, an often-cited barrier to Black participation in research often felt by the African American community, it is important to explore the multitude of ethnicities factored into the grouping of Black and the variance of experience impacting attribution. This study explores barriers to participation expressed by those who were engaged directly for this study, who span a multitude of ethnicities.

In terms of research specifically examining barriers to research participation for African and Caribbean communities, the literature is extremely scarce. In fact, the existent studies in the literature are examining the UK, where there are large African and Caribbean communities. This is a noteworthy deficiency for US studies, as the USA also has large African and Caribbean populations. However, the studies from the UK have rendered some interesting results that are also relevant for this study. In working to recruit African and Caribbean men with prostate cancer and their partners for qualitative research to explore their psychosocial needs after prostate cancer (CaP) treatment and how best to address them, Bamidele et al. found that gatekeeping and building rapport were extremely important as establishing trust with potential participants through rapport building was perceived to also have enhanced successful recruitment for this study. For this study, in an effort to build rapport, the same researcher followed potential participants from recruitment to conducting their interviews, which fostered a sense of familiarity with participants and encouraged a high retention rate as well, as all the participants recruited were successfully interviewed [15]. Bamidele et al. also found value in the importance of the researcher’s ethnicity, as they found that the usage of face-to-face interviews, coupled with the interviewer’s African ethnicity, may have positively impacted rapport building with study participants as many of them seemed to identify with her as an “insider” [15].

The concept of cultural beliefs as a barrier to research participation has minimally been examined. In a study examining how acceptable it would be for HIV-positive women who identified as African, Caribbean, or African American to provide breastmilk and fluid samples for research, the finding that some of the respondents would find it unacceptable to provide breast milk/fluid samples was closely connected to their cultural beliefs that that breast milk/fluids can be used as a medium for witchcraft, and this belief was regarded by researchers as a potential barrier [16]. An examination of barriers to participation from culturally and linguistically diverse individuals in clinical trials uncovered that beliefs about health and health care can vary significantly according to different cultures and may clash with mainstream norms, making individuals from these backgrounds less likely to explore all the healthcare options available to them [17], such as screening.

Religious faith, which is quite intertwined with culture, was found to encourage screening participation for Black African and Black Caribbean participants in one study, concluding that participants of either Christian or Muslim beliefs felt that screening was seen as a way of helping oneself [18]. Overall, faith in God encouraged screening participation for Black African and Black Caribbean participants from varying religious backgrounds [18], a finding that is important to the present study.

While barriers in the literature are frequented, the literature does also highlight some successful strategies to recruit within the Black community. Williams and Anderson (2021) found that partnerships between academic research centers and community health workers (CHW’s) are essential. They morph the definition of CHW’s to include community-based partners such as local department of health and wellness promotion, community centers, and social services, and note that CHW’s play a key role as liaisons in facilitating access to participants [19]. The CHW’s already have established and trusted relationships, between the family unit and the health care system through an individual that Williams and Anderson label the “kin keeper.” Kin keepers for their study met the following criteria: (1) participant in a CHW’s non-cancer-related public health program (e.g., maternal and child health); (2) self-identified in one of the three racial/ethnic groups; (3) able to recruit, in any combination, 2–4 adult bloodline female family members (mother, sister, daughter, grandmother, aunt) to participate; and (4) willing to assist the CHW’s in locating the family members for the 12-month follow-up visit [20]. The second effective recruitment strategy identified by Williams and Anderson was leveraging membership organizations and events. Other studies have identified more communications-based strategies working effectively. A health education research study found gender differences in successful recruiting in the Black community. While recruiting for a 2-year randomized behavioral health education intervention, researchers found that radio ads were the predominant way men were successfully recruited, while significantly more women indicated hearing about the study via TV news stories or social media than men [21]. Other studies consistently cite community advertisements, local churches, historically black sororities and other social networks, and institution-based participant resource pools as successful strategies utilized [22]. Field-based strategy and snowballing are also often cited [23].

This paper will examine effective recruitment strategies in Black communities of New York City that were utilized during data collection for part of a larger study examining colorectal cancer screening behaviors and intent.

## Methods

The main investigator/recruiter was an African-American woman who leveraged current relationships and built new ones to aid with recruitment. She was accurately perceived and welcomed as a member of the community genuinely passionate about the health of the Black community, and this was critical for recruitment. Barber shops and hair salons were targeted based on the recruiter’s past success with recruitment in these settings; they are viewed as safe communal places in the Black community that have also been documented as successful sites for health promotion [24].

### Recruitment Procedures

An intentional two-part recruitment process was utilized for this study. The first part allowed for 3 months of engagement in target neighborhoods of New York City boroughs. This stage involved building new and strengthening pre-existing relationships with churches, barbershops, community centers, and businesses in areas with significant Black populations. The primary researcher established solid trusting relationships with the gatekeepers of these businesses, which enabled the gatekeepers to experience the honesty, sincerity, and passion of the researcher in terms of a solid interest in advancing the health of the Black community. In turn, the gatekeepers conveyed to their populations their own trust in the researcher and the work, and consequently urged others to participate in the study. Within the churches, salons, and barbershops, interested individuals were prescreened for eligibility in person. Eligibility criteria included adults: (1) ages 18 years and over; (2) self-identified as Black; and (3) expressed willingness to complete a survey that included an informed consent form and authorization for future use of the data. Interested and eligible individuals then completed the survey at that time. All participants completed the study survey on a freshly sanitized tablet (paper copies were also available) and participants were compensated $5 in cash for completion of the survey.

The same primary researcher established a regular visitation schedule to the participating sites which strengthened rapport with the gatekeepers, built and established visibility and consistency with the populations, and enabled trust to consistently grow among potential participants. The mere exposure effect holds that repeated exposure to a stimulus can lead individuals to consider the stimulus as more pleasant [25]. Individuals will have a preference or liking for things they have repeatedly been exposed to, or at least that familiarity breeds liking more than contempt [26], which was evidenced by comments that gatekeepers informally shared with the researcher (Table 1). Some individuals who initially declined participation with explanations of (1) lack of time, (2) lack of interest, and (3) mistrust of the institution backing the study moved to a place of (1) having time, (2) becoming interested when seeing their counterparts engage and participate, and (3) being less concerned with the backing institution, and more concerned with the trustworthiness of the primary researcher who they were regularly seeing and engaging with.

**Table 1:** Feedback informally provided to the researcher by gatekeepers and participants

Topic	Feedback	Source
Mistrust	At first, I didn’t know what you were doing here—all I knew was that I usually come to the barbershop to get a shape up, and to relax. I didn’t want any part of what you had going on. But then, you were here at least 2–3 other times when I was here and after I saw everyone vibing with you, I decided to check you out. I’m glad I did, and I hope this helps Black men in our area	Participant
Mistrust	You’ve gotten participation from people I didn’t think would do it. You’ve been here week after week and my clients see that. It makes a difference sis	Gatekeeper
Appreciation	We’re so thankful for your faithfulness to our church and our community, particularly in a pandemic. It is invaluable to have one of us, care about our health	Gatekeeper
Compensation	I don’t even care about that. We should be paying you for doing things in this area that we need	Participant

The first part involved registering individuals who were interested in participating in the study and consented to be contacted and reminded to come to the location where they were recruited on an actual date to complete the actual survey.

The second part of the recruitment process utilized availability sampling outside of NYC subway stations and other high traffic areas as well as large community events. This part of the recruitment process took place once New York City began to open up after the COVID-19 shutdown. We targeted high traffic subway stations such as Atlantic Terminal-Barclays Center (Brooklyn), 125th Street (West Harlem), Flatbush Avenue (Brooklyn), 125th Street (East Harlem), 116th Street (Harlem), and 149th-Grand Concourse (Bronx). These stations not only have a very high traffic volume [27], but they are also in areas with large Black populations. Additionally, as events were slowly beginning to take place in New York City again, we utilized street festivals and community events on Flatbush Avenue (Brooklyn), Fulton Street (Brooklyn), and W. 116th and W. 125 Streets in Harlem. Lastly, we visited areas such as Morningside Park (Harlem), Marcus Garvey Park (Harlem), Prospect Park (Brooklyn), and cafes and barbershops in Bedstuy Brooklyn, and Flatbush/Little Caribbean Brooklyn.

## Data Analysis

Data was analyzed using SPSS Statistics. Descriptive statistics were run for all demographic variables for participants. Key recruitment strategies that were documented throughout study implementation were systematically reviewed with team members and reflected upon. Field notes were kept by the primary researcher, as well as feedback given to the primary researcher by the gatekeepers and participants. There are 504 subjects in the original data set. Then, we deleted seven (7) subjects for missing cases, leaving us with a total of 497. The demographic characteristics of the *n* = 497 subjects included in the analysis set are summarized in Table 2.

**Table 2:** Descriptive characteristics of participants

	Frequency	% Frequency
Ethnicity		
African-American	404	38.4%
Caribbean	87	14.5%
African	6	9.3%
Gender		
Male	229	46.1%
Female	261	52.5%
Neutral	1	0.2%
Non-binary	3	0.6%
Queer	2	0.4%
Female transgender	1	0.2
Area		
Brooklyn	178	35.8%
Manhattan	287	57.7%
Bronx	24	4.8%
Long Island	2	0.4%
New Jersey	3	0.6%
Age		
18–24	65	13.1%
25–34	110	22.1%
35–44	85	17.1%
45–54	79	15.9%
55–64	115	23.1%
65–74	38	7.6%
75–84	4	0.8%
85 +	1	0.2%
Income		
Under $20,999	191	38.4%
$ 21,000–$30,999	72	14.5%
$31,000–$40,999	46	9.3%
$41,000–$50,999	40	8.0%
$51,000–$60,999	42	8.5%
$61,000–70,999	25	5.0%
$71,000–$80,999	21	4.2%
$81,000–$90,999	11	2.2%
$91,000–$100,999	16	3.2%
$101,000–$150,999	22	4.4%
Above $151,000	11	2.2%
Marital status		
Single	328	66.0%
Married	86	17.3%
Divorced	23	4.6%
Widowed	19	3.8%
Separated	12	2.4%
Living with partner	29	5.8%
Education		
8th Grade	6	1.2%
High school graduate	89	17.9%
Some high school	111	22.3%
Some college	37	7.4%
Trade school	9	1.8%
Bachelor’s degree	97	19.5%
Master’s degree	43	8.7%
Associates degree	61	12.3%
Doctoral degree	11	2.2%
Employment status		
Part time	73	14.7%
Full time	202	40.6%
Seasonal	19	3.8%
Not working, retired	203	40.8%

*N* = 497

## Results

In part one of the recruitment process, we attempted to engage with 488 Black-American men and women between November 2019 and February 2020. Of this number, *n* = 29/0.06% declined to participate due to a lack of time, *n* = 11/0.02% declined due to not trusting institutions conducting research, and *n* = 24/0.04% declined to participate due to the incentive not being enough to compensate for their time. When data collection resumed in the late summer of 2020 after being abruptly halted due to COVID-19, of the 424 who consented to be recontacted, *n* = 33 individuals (78%) were still interested and agreed to come to the sites to participate; *n* = 42/0.099%) could not be reached and *n* = 51/12% were uncomfortable coming out to their original recruitment site due to COVID. A total of those both engaged prior to the COVID-19 shutdown (*n* = 331) and those newly engaged in the late summer of 2020 within the same recruitment sites (*n* = 37) rendered a total of *n* = 368 individuals who completed the survey.

In the second part of the recruitment process, we engaged with 319 individuals (*n* = 319). Of these eligible participants, *n* = 83/26% declined to participate due to a lack of time, *n* = 61/19% declined to participate due to the incentive not being enough to compensate for their time, *n* = 27/0.084% did not feel comfortable understanding the questions due to a language barrier, and *n* = 12/0.038% declined due to not trusting institutions conducting research. A total of 43% of those engaged (*n* = 136) completed the survey through this method.

Of the total engaged (*n* = 807), *n* = 112 individuals (14%) declined to participate due to a lack of time. Most were rushing to their destination and did not really hear what the study was about. Some received information about the study and then ascertained that they did not have the time to complete a questionnaire. A lack of time was the major reason that individuals declined to participate. The second major reason was that the incentive was not enough to compensate for their time (*n* = 85/11%). Additionally, of the total engaged (*n* = 807), 0.02% (*n* = 23) declined due to not trusting institutions conducting research, and 0.03% (*n* = 27) did not feel comfortable understanding the questions due to a language barrier (Table 3).

**Table 3:** Barriers to participation

Barriers to participation	Part one	Part two
Original engaged	488	319
Lack of time	29	83
Distrust of institutions conducting research	11	12
Incentive not enough to compensate for their time	24	61
Uncomfortable understanding the questions due to a language barrier	0	27
Uncomfortable coming out to the recruitment site due to COVID	51	N/A
Could not be reached	42	N/A
Engaged after the city reopened post shutdown	37	N/A
Total surveys completed	368	136

*N* = 504

## Discussion and conclusion

The two-tiered model of recruitment that involved consistent engagement and having the primary researcher lead this recruitment process was extremely beneficial. Trust was established and relationships were forged within pillars of the communities. While the halt due to COVID-19 was initially a major concern for the research team, it actually strengthened the relationship between the business owners/church leaders/community center leaders and the research team as the primary investigator maintained contact throughout the pandemic shutdown and immediately after. Through the months of engagement that went to 3 months short of a year, relationships were built; community leaders were able to see that the concern and care form the researcher for the community and its health was genuine, and not solely tied to study completion. This dynamic also ensured community leaders that what they feared, researchers coming into the community, getting data they desired and leaving, would not be the outcome. Additionally, the second part of the recruitment model that relied more heavily on street canvassing and availability unveiled that a lack of time and a small incentive to value participant’s time were the two major concerns. This study allowed for a small incentive ($5), which would definitely be adjusted in future studies. Some expressed that they felt that only the Black communities were compensated at such low rates for their time which should certainly be assessed and addressed. The survey took about 15 min to complete, based on both an estimate from the survey tool Qualtrics, and the research team doing a few trial completions of the survey. This time was quoted verbally to potential participants, and also was listed on the consent form. Mistrust of researchers was still a concern; however, it may have been lower as a significant percentage of individuals engaged were in largely Caribbean and African communities. Kapiriri et al. (2017) noted their surprise that their respondents did not identify historical experiences with research as a reason for their reluctance to participate in their study as it is a barrier noted throughout the literature. Their conclusion was that as most of the respondents were African and Caribbean immigrants, they may not be aware of the negative history of research on Black populations in North America [16]. As this study is conducted in the USA, we find it more plausible that these communities are aware of the negative history, but may be less connected to that history. As the primary researcher spent so much time in the churches engaging with church and community members, it was uncovered that the church is most certainly a vessel promoting that participation is research so that researchers can advance their findings related to Black communities, and also so that the communities can in turn hopefully benefit from this research in the form of improved community health.

Research endeavors in more historic African American communities like Harlem interestingly experienced large participation. In the Caribbean and African communities, researchers experienced barriers where individuals did not feel confident in their ability to understand the questions being asked. Also, it was interesting that the respondents who expressed concern about their confidence in their ability to understand the survey questions only did so in part two of the recruitment process (street canvassing), which led the research team to believe that perhaps there was more comfort and/or support felt in the settings where consistent engagement had been taking place in part one of the recruitment, and where relationships were built. Respondents may have felt that they could comfortably ask for help in that setting versus the street canvassing setting. For future research, it would be helpful to have a creole version of a survey available as a large number of individuals who expressed this concern were Haitian creole speaking, and otherwise wanted to participate. Extracting within race differences was pivotal in demystifying the conclusion of numerous studies that African Americans specifically are hesitant to participate due to historical mistrust related to tragedies such as the Tuskegee Experiment and numerous other occurrences of African Americans being treated as guinea pigs for the advancement of research. Mistrust in this study primarily came from individuals we approached in the Caribbean neighborhoods, where many expressed more comfort with home remedies and bush doctors when asked about colorectal cancer screening and declined to participate. They expressed a disdain for not just research, but going to the doctor for colorectal screening as opposed to relying on home remedies and those of their bush doctors. It was informally determined that over 50 individuals (some who participated and some who did not) expressed in conversation with the primary researcher that they were more comfortable with home remedies and bush doctors than clinical visits and screening. Once the primary researcher’s informal count reached 50, it was determined that this topic alone is a viable study that should be further explored. Innovative communication, qualitative research, and recruitment strategies tailored to the Caribbean population are needed in future studies to address this recruitment challenge that we experienced.

There were some limitations to this study. The researcher had a sheet with the options of (1) lack of time, (2) incentive not enough to compensate for their time, (3) distrust of institutions conduction research, and (4) not comfortable understanding the questions due to a language barrier. One of these options was marked for individuals who declined to participate, after they either expressed their reasoning or were probed. It would have been great to capture sex of the individuals, to see if a particular gender was more prone to be interested. That was not necessarily the focus but could have added some great information. Another limitation could have been that this study had solely a Black woman in the field. While the study was about colorectal cancer screening and not something more sensitive to many men like prostate cancer, the gender of the researcher may have made a difference for some if a male was also recruiting. In all honesty, very few men were not open to engaging, and for those who were, it was attributed to a lack of time. We also do not have the ethnicity of all of the individuals who we simply engaged with. That data would have added richness to this paper; however, it would have also been difficult to obtain while many who declined were in a rush, or skeptical of providing demographic information. Lastly, a lot could be gathered on this topic in a qualitative data collection setting, allowing community members who identify as Black but represent multiple ethnicities, the opportunity to discuss barriers first hand.

In summation, research needs to dive deeper into barriers to recruitment when summarizing these barriers as those of individuals who identify as Black. In New York City, those we engaged with who identified as Black were African American, Jamaican, Trinidadian, Haitian, Panamanian, Nigerian, Ghanaian, Guinean, and the list goes on. Many identified as being bi-ethnic. The barriers to recruitment of individuals of these ethnicities and many others are varied and deserve to be explored and understood in future research. Future research will ensure a qualitative voice, and survey adjustments and tailoring.

## Data Availability

The data analyzed during the current study is available from the corresponding author on reasonable request.
